# Characteristics of 1270 Chinese sibling pairs with cancer

**DOI:** 10.1186/s12885-021-08737-0

**Published:** 2021-09-15

**Authors:** Ju Liu, Jian Yin, Yiwei Liu, Zhijian Xu, Kai Zhang

**Affiliations:** 1 Department of Cancer Prevention, National Cancer Center/National Clinical Research Center for Cancer/Cancer Hospital, Chinese Academy of Medical Sciences and Peking Union Medical College, 17 South Panjiayuan Lane, Chaoyang District, Beijing, 100021 P. R. China; 2 Department of Cancer Epidemiology, National Cancer Center/National Clinical Research Center for Cancer/Cancer Hospital, Chinese Academy of Medical Sciences and Peking Union Medical College, Beijing, 100021 P.R. China; 3 School of Population Medicine and Public Health, Chinese Academy of Medical Sciences and Peking Union Medical College, Beijing, 100730 China; 4 College of Life Sciences, Hebei University, Baoding, 071002 P.R. China

**Keywords:** Cancer, Family history, Sibling, Screening, Malignant disease, Genetic service

## Abstract

**Background:**

Previous research found that the cancer history of an individual’s sibling may be a better indicator than that of the parents. We aim to provide recommendations for opportunistic screening for individuals whose sibling had been diagnosed with cancer.

**Methods:**

During the physical examination in Cancer Hospital, Chinese Academy of Medical Sciences, 43,300 people were asked if they have at least two siblings who developed cancer.

**Results:**

A total of 1270 sibling-pairs from 766 families developed cancer, including 367 pairs of brothers (Bro-pairs), 368 pairs of sisters (Sis-pairs), and 535 pairs of brother-and-sister (BroSis-pairs). The mean ages at diagnosis of cancer for the three groups were from 58 to 62 years. More than half of Bro-pairs (55.3%) or Sis-pairs (51.1%) had cancer from the same systemic origin, and more than a quarter of Bro-pairs (28.1%) and Sis-pairs (37.2%) developed the same type of cancer. However, only 36.0% of BroSis-pairs developed cancers from the same systemic origin, and 18.9% developed the same type of cancer. In Bro-pairs and BroSis-pairs, lung cancer and digestive system cancer were the most common cancers, while in Sis-pairs, breast cancer, lung cancer, cervical cancer, liver cancer and thyroid cancer were the most common ones.

**Conclusions:**

If an individual’s sibling is diagnosed with cancer, the individual should consider participating in opportunistic screening annually, especially for lung cancer and digestive system cancers for both sexes. For sisters, breast cancer, cervical cancer and thyroid cancer should be screened early. Additionally, genetic services are essential for individuals who have siblings with cancer.

## Introduction

Cancer incidence and mortality are rapidly growing worldwide [1]. There is increasing attention to cancer prevention and early detection programs, especially among people with a family history of cancer. Family history is a strong indicator for evaluating cancer risks [2, 3], as people with a family history of cancer have a significantly higher risk of developing cancer than the general population [4, 5].

Familial cluster data reveals that there is important interaction between inherited genes and shared environmental factors, and cancer outcomes. Friedman et al. indicated that the siblings of long-term childhood cancer survivors have an increased risk of cancer [6]. Similar to parents and their children, siblings share genetic and environmental factors. However, comparing to the case of parents and children, siblings are more likely to develop similar lifestyle and dietary habits, especially siblings of the same sex. Therefore, the risk of cancer of an individual is more strongly associated with the cancer history of the siblings rather than the parents [5]. However, among Chinese population, few studies investigated and analyzed the characteristics of siblings with cancers.

The aim of our study is to evaluate the probability of siblings developing the same cancer types or cancers from the same systemic origin, and to assess their age of diagnosis. We also aim to develop recommendations on opportunistic screening for early detection of cancer for individuals whose siblings have histories of cancers.

## Materials and methods

### Study population

During their physical examination visit to the Department of Cancer Prevention at the Cancer Hospital of the Chinese Academy of Medical Sciences from January 2008 to December 2019, 43,300 individuals were asked if they have at least two siblings (including themselves) who had been diagnosed with cancer. The diagnosed age (year) and cancer types of siblings need to be confirmed again by calling to first degree other family member by participant. Participants were included in our study if (1) two or more siblings from the same biological parents had been diagnosed with cancer, and (2) the siblings’ ages at diagnosis of cancer were available. The excluding criteria include: (1) primary cancer sites of any family member were unknown; (2) age of any family member with cancer at diagnosis was unavailable; (3) The participant’s father or mother was diagnosed with cancer. (4) Participants had long-term occupational exposure. A total of 1270 sibling pairs from 766 families were included in the study.

### Statistical analysis

In this study, cancer from the same systemic origin includes digestive system cancer, reproductive system cancer, respiratory system cancer, endocrine system cancer, circulatory system cancer, and urinary system cancer. Digestive system cancer includes tongue cancer, esophageal cancer, stomach cancer, colorectal cancer, liver cancer, pancreatic cancer and gallbladder cancer. Reproductive system cancer includes breast cancer, endometrial cancer, ovarian cancer, cervical cancer, prostate cancer, testicular cancer and vulvar cancer. Respiratory system cancer includes nasopharyngeal cancer, laryngeal cancer and lung cancer. Endocrine system cancer includes thyroid cancer. Circulatory system cancer includes malignant lymphoma, leukemia and multiple myeloma. Urinary system cancer includes kidney cancer, ureter cancer and bladder cancer. Differences between ages of diagnosis of siblings in sibling-pairs were calculated and analyzed with one-way ANOVA test or independent student’s t-test. Categorical variables were presented as number (percentage) and were compared using the chi-square test. Data analyses were conducted with SPSS software package, version 16.0 (SPSS Inc., Chicago, IL, USA).

## Results

### General information

Among 766 families with two or more siblings diagnosed with cancer, 586 (76.5%) families had 2 siblings with cancer, 143 (18.7%) had 3, 29 (3.8%) had 4, 7 (0.9%) had 5, and 1 (0.1%) had 6. These siblings were divided into sibling pairs. There were 367 pairs of brothers (Bro-pairs) from 216 families, 368 pairs of sisters (Sis-pairs) from 237 families, and 535 pairs of brother-and-sister (BroSis-pairs) from 313 families.

### Cancer types

The proportions of cancer diagnosis in Bro-pairs, Sis-pairs and BroSis-pairs are shown in Table 1. As expected, lung cancer was the most common cancer among Bro-pairs and male from BroSis-pairs. For Sis-pairs and female from BroSis-pairs, breast cancer was the most common. The 5 most common cancer types in Bro-pairs were lung cancer, stomach cancer, liver cancer, colorectal cancer and esophageal cancer, which was consistent with male from BroSis-pairs. For Sis-pairs, the five most common cancer types were breast cancer, lung cancer, colorectal cancer, thyroid cancer and liver cancer. The result was also consistent with females in the BroSis-pairs, except the fourth most common cancer type being stomach cancer instead of thyroid cancer.

**Table 1:** Top ten cancers stratified by sibling-groups

No.	Brothers		Sisters		Brothers and sisters					
					Brothers		Sisters		Both	
	Disease	N (%)	Disease	N (%)	Disease	N (%)	Disease	N (%)	Disease	N (%)
1	Lung cancer	146 (26.5)	Breast cancer	210 (35.4)	Lung cancer	126 (28.2)	Breast cancer	103 (23.1)	Lung cancer	224 (25.1)
2	Stomach cancer	92 (16.7)	Lung cancer	109 (18.3)	Stomach cancer	72 (16.1)	Lung cancer	98 (22.0)	Stomach cancer	112 (12.5)
3	Liver cancer	85 (15.5)	Colorectal cancer	55 (9.3)	Liver cancer	58 (13.0)	Colorectal cancer	55 (12.3)	Colorectal cancer	107 (12.0)
4	Colorectal cancer	66 (12.0)	Thyroid cancer	31 (5.2)	Colorectal cancer	52 (11.7)	Stomach cancer	40 (9.0)	Breast cancer	104 (11.7)
5	Esophageal cancer	58 (10.5)	Liver cancer	28 (4.7)	Esophageal cancer	41 (9.2)	Liver cancer	25 (5.6)	Liver cancer	83 (9.3)
6	Pancreatic cancer	19 (3.5)	Ovarian cancer	28 (4.7)	Pancreatic cancer	16 (3.6)	Endometrial carcinoma	20 (4.5)	Esophageal cancer	55 (6.1)
7	Kidney cancer	12 (2.2)	Endometrial carcinoma	27 (4.5)	Prostate cancer	13 (2.9)	Pancreatic cancer	16 (3.6)	Pancreatic cancer	32 (3.6)
8	Malignant lymphoma	12 (2.2)	Stomach cancer	25 (4.2)	Bladder cancer	12 (2.7)	Esophageal cancer	14 (3.1)	Endometrial carcinoma	20 (2.2)
9	Leukemia	11 (2.0)	Pancreatic cancer	18 (3.0)	Kidney cancer	8 (1.8)	Cervical cancer	14 (3.1)	Thyroid cancer	18 (2.0)
10	Laryngeal cancer/Prostate cancer^a^	7 (1.3)	Cervical cancer	13 (2.2)	Gallbladder cancer/Malignant lymphoma^a^	7 (1.6)	Ovarian cancer	13 (2.9)	Bladder cancer	15 (1.7)
Sum		508 (92.4)		544 (91.6)		405 (90.8)		398 (89.2)		770 (86.3)

^a^The number of Laryngeal Cancer, Prostate Cancer, Gallbladder Cancer, and Malignant Lymphoma were all 7

### Age of diagnosis

The mean age of cancer diagnosis was 61.8 ± 12.1 for Bro-pairs, 57.8 ± 12.2 for Sis-pairs, and 60.9 ± 11.6 for BroSis-pairs. For sis-pairs, the mean age of diagnosis is significantly younger than the other two groups (both *P* < 0.001).

In each group (Bro-pair, Sis-pair and BroSis-pair), the 30-year range of age of diagnosis with the highest percentage of diagnosis was analyzed, according to the data from Table 2. In Bro-pairs, 80.2% of brothers developed cancer between the age of 46 and 75. In Sis-pairs, 75.4% of sisters developed cancer between 41 and 70. In BroSis-pairs, 83.7% of brothers developed cancer between 46 and 75, while 79.3% of sisters developed cancer during the same 30-year range.

**Table 2:** Age of diagnosis stratified by sibling-groups

Age	Brothers N (%)	Sisters N (%)	Brother-and-sister N (%)			All N (%)
			Brothers	Sisters	Both	
≤ 30	5 (0.9)	7 (1.2)	3 (0.7)	4 (0.9)	7 (0.8)	19 (0.9)
31 ~ 35	8 (1.5)	16 (2.7)	9 (2.0)	4 (0.9)	13 (1.4)	37 (1.8)
36 ~ 40	14 (2.5)	24 (4.0)	5 (1.1)	22 (4.9)	27 (3.0)	65 (3.2)
41 ~ 45	24 (4.4)	61 (10.3)	20 (4.5)	26 (5.8)	46 (5.2)	131 (6.4)
46 ~ 50	53 (9.6)	83 (14.0)	32 (7.2)	48 (10.8)	80 (9.0)	216 (10.6)
51 ~ 55	66 (12.0)	72 (12.1)	70 (15.7)	57 (12.8)	127 (14.2)	265 (13.0)
56 ~ 60	85 (15.5)	89 (15.0)	85 (19.1)	53 (11.9)	138 (15.5)	312 (15.3)
61 ~ 65	79 (14.3)	83 (14.0)	65 (14.6)	72 (16.2)	137 (15.4)	299 (14.7)
66 ~ 70	86 (15.6)	60 (10.1)	62 (13.9)	67 (15.0)	129 (14.5)	275 (13.5)
71 ~ 75	72 (13.1)	56 (9.4)	59 (13.2)	57 (12.8)	116 (13.0)	244 (12.0)
76 ~ 80	34 (6.2)	30 (5.0)	20 (4.5)	24 (5.4)	44 (4.9)	108 (5.3)
81 ~ 85	15 (2.7)	11 (1.9)	10 (2.2)	10 (2.2)	20 (2.2)	46 (2.3)
86 ~ 90	8 (1.5)	2 (0.3)	6 (1.3)	1 (0.2)	7 (0.8)	17 (0.9)
> 90	1 (0.2)	0 (0)	0 (0)	1 (0.2)	1 (0.1)	2 (0.1)
Total	550 (100)	594 (100)	446 (100)	446 (100)	892 (100)	2036 (100)

The 5-year range of age of diagnosis with the highest percentage of diagnosis for bro-pairs was 66–70 years old, comparing to 56–60 years old for the Sis-pair group. For the BroSis-pairs, the range was 56–60 for brothers, and 61–65 for sisters. However, it should be noted that within the bro-pairs, there is only 1 less case within the 56–60 years old group comparing to the 66–70 years old group (Table 2).

### Category of cancer from systemic origins

As shown in Tables 3, 203 out of 367 (55.3%) Bro-pairs were diagnosed with cancers from the same systemic origin (same-origin cancers). The digestive system cancer (157, 77.3%) was the most common. The second most common was respiratory system cancer (44, 21.7%), while cancer from other cancer origins only accounted for 1% (2 cases). Out of 368, 188 (51.1%) Sis-pairs were diagnosed with the same-origin cancers, which was slightly lower than Bro-pairs. Among sis-pairs, the reproductive system cancer (114, 60.6%) was the most common, followed by digestive (36, 19.1%), respiratory (32, 8.7%) and endocrine (6, 3.2%) system cancers. Only 192 pairs out of 535 (36.0%) BroSis-pairs were diagnosed with same-origin cancers, which was significantly lower than both Bro-pair (*P* < 0.001) and Sis-pair (*P* < 0.001). Among the BroSis-pairs, the most common one was digestive system cancer (126, 65.6%), followed by respiratory system cancers (51, 26.6%), reproductive system cancers (10, 5.2%) and other cancer origins (5, 2.6%).

**Table 3:** Sibling-pairs with cancers from the same systemic origin, stratified by sibling-groups

	Same system N (%)			
	Brother	Sisters	Brother-and-sister	All
Digestive system	157 (77.3)	36 (19.2)	126 (65.6)	319 (54.7)
Respiratory system	44 (21.7)	32 (17.0)	51 (26.6)	127 (21.8)
Reproductive system	1 (0.5)	114 (60.6)	10 (5.2)	125 (21.5)
Blood system	1 (0.5)	0	1 (0.5)	2 (0.3)
Endocrine system	0	6 (3.2)	3 (1.6)	9 (1.5)
Urinary system	0	0	1 (0.5)	1 (0.2)
Sum (A/B)^a^	203/367 (55.3)	188/368 (51.1)	192/535 (36.0)	583/1270 (45.9)

^a^ A, the sum of the sibling-pairs with cancers from same systemic origin; B, the sum of the sibling-pairs with cancers

### Types of cancer

As shown in Table 4, among 367 Bro-pairs, 103 (28.1%) were diagnosed with the same cancer type. The most common one was lung cancer, followed by liver cancer, stomach cancer, esophageal cancer and colorectal cancer. Among 368 Sis-pairs, 137 (37.2%) were diagnosed with the same cancer type. The most common ones were breast cancer, lung cancer, cervical cancer, liver cancer, thyroid cancer, and colorectal cancer. For BroSis-pairs, only 101 (18.9%) pairs developed the same type of cancer. The 5 most common cancer types were identical as the Bro-pairs. The proportion of the same cancer type of Sis-pairs was the highest, followed by Bro-pairs (*P* = 0.010) and BroSis-pairs (*P* < 0.001).

**Table 4:** Sibling-pairs with the same type of cancer, stratified by sibling-pairs

No.	Brothers		Sisters		Brother-and-sister	
	Disease	N (%)	Disease	N (%)	Disease	N (%)
1	Lung Cancer	37 (35.9)	Breast Cancer	77 (56.2)	Lung Cancer	46 (45.5)
2	Liver Cancer	22 (21.4)	Lung Cancer	29 (21.2)	Stomach Cancer	15 (14.8)
3	Stomach Cancer	19 (18.4)	Cervical Cancer	7 (5.1)	Colorectal Cancer	14 (13.9)
4	Esophageal Cancer	13 (12.6)	Liver Cancer	5 (3.6)	Liver Cancer	11 (10.9)
5	Colorectal Cancer	9 (8.7)	Thyroid Cancer	5 (3.6)	Esophageal Cancer	6 (5.9)
6	Nasopharyngeal Carcinoma	1 (1.0)	Colorectal Cancer	5 (3.6)	Thyroid Cancer	3 (3.0)
7	Prostate Cancer	1 (1.0)	Ovarian Cancer	4 (2.9)	Pancreatic Cancer	3 (3.0)
8	Lymphoma	1 (1.0)	Esophageal Cancer	2 (1.5)	Breast Cancer	1 (1.0)
9	–	–	Pancreatic Cancer	2 (1.5)	Myeloma	1 (1.0)
10	–	–	Stomach Cancer	1 (0.7)	Cholangiocarcinoma	1 (1.0)
Sum (A/B)^a^		103/367 (28.1)		137/368 (37.2)		101/535 (18.9)

^a^A, the sum of the sibling-pairs with same type of cancer; B, the sum of the sibling-pairs with cancer

### Differences between ages of diagnosis

For each group, differences between ages of diagnosis of two siblings (age differences) were calculated (Table 5). The most common age-difference group for all three sibling-pair groups was 1–5 years. For Bro-pairs, this age-difference group accounted for 30.8% of cases, while the percentage was comparable for sis-pairs (32.6%) and BroSis-pairs (30.1%), with no significant difference between groups (all *P* > 0.05). When considering the age differences within 10 years, the result across sibling-pair groups were also not significant (all *P* > 0.05) difference, with 224 (61.1%) in Bro-pairs, 231 (62.8%) in Sis-pairs and 334 (62.4%) in BroSis-pairs. For all sibling-pair groups, more than three quarters developed diseases within 15 years of age differences (79.0% for Bro-pairs, 76.3% for Sis-pairs, 77.9% for BroSis-pairs).

**Table 5:** Differences in age of diagnosis to systemic origins of cancers, stratified by sibling-pair groups

Age difference	Brother N (%)			Sister N (%)			Brother-and-sister N (%)			All N (%)		
	Same system	Different system	Both	Same system	Different system	Both	Same system	Different system	Both	Same system	Different system	Both
0	16 (7.9)	14 (8.5)	30 (8.2)	17 (9.0)	13 (7.2)	30 (8.2)	14 (7.3)	17 (5.0)	31 (5.8)	47 (8.1)	44 (6.4)	91 (7.2)
1–5	66 (32.5)	47 (28.7)	113 (30.8)	70 (37.2)	50 (27.8)	120 (32.6)	59 (30.7)	102 (29.7)	161 (30.1)	195 (33.4)	199 (29.0)	394 (31.0)
6–10	47 (23.1)	34 (20.7)	81 (22.1)	44 (23.4)	37 (20.6)	81 (22.0)	52 (27.1)	90 (26.2)	142 (26.5)	143 (24.5)	161 (23.4)	304 (23.9)
11–15	38 (18.7)	28 (17.1)	66 (18.0)	21 (11.2)	29 (16.1)	50 (13.6)	30 (15.6)	53 (15.5)	83 (15.5)	89 (15.3)	110 (16.0)	199 (15.7)
16–20	15 (7.4)	15 (9.1)	30 (8.2)	21 (11.2)	20 (11.1)	41 (11.1)	16 (8.3)	37 (10.8)	53 (9.9)	52 (8.9)	72 (10.5)	124 (9.8)
21–25	12 (5.9)	12 (7.3)	24 (6.5)	10 (5.3)	17 (9.5)	27 (7.3)	11 (5.7)	21 (6.1)	32 (6.0)	33 (5.7)	50 (7.3)	83 (6.5)
26–30	5 (2.5)	7 (4.3)	12 (3.2)	3 (1.6)	6 (3.3)	9 (2.5)	7 (3.7)	14 (4.1)	21 (3.9)	15 (2.6)	27 (3.9)	42 (3.3)
> 30	4 (2.0)	7 (4.3)	11 (3.0)	2 (1.1)	8 (4.4)	10 (2.7)	3 (1.6)	9 (2.6)	12 (2.3)	9 (1.5)	24 (3.5)	33 (2.6)
Total	203 (100)	164 (100)	367 (100)	188 (100)	180 (100)	368 (100)	192 (100)	343 (100)	535 (100)	583 (100)	687 (100)	1270 (100)

For cancer from the same systemic origin (Table 5), 69.6% of Sis-pairs developed diseases within 10 years of age differences, which was significantly higher than cancer from different systemic origins (55.6%, *P* = 0.005). For Bro-pairs, 63.5% developed same-origin cancers within 10 years of age differences, which was not significantly different than bro-pairs who developed different-origin cancers (57.9%, *P* > 0.05). Similarly, 65.1% of BroSis-pairs developed same-origin cancers within 10 years of age differences, which was not significantly different than BroSis-pairs who developed different-origin cancers (60.9%, *P* > 0.05).

When considering cancer types (Table 6), for age differences less than 10 years, the proportion of all sibling-pair groups who developed same cancer types were higher than the groups who developed different cancer types (66.1% vs 59.0% for Bro-pair, 69.1% vs 59.1% for Sis-pairs, and 70.3% vs 60.6% for BroSis-pair), but these differences were not significant (all *P* > 0.05).

**Table 6:** Difference in age of diagnosis to cancer types, stratified by sibling-pair groups

Age difference	Brother N (%)		Sister N (%)		Brother-and-sister N (%)		All N (%)	
	Same cancer	Different cancer	Same cancer	Different cancer	Same cancer	Different cancer	Same cancer	Different cancer
0	8 (7.8)	22 (8.3)	13 (9.5)	17 (7.4)	8 (7.9)	23 (5.3)	29 (8.5)	62 (6.8)
1–5	38 (36.9)	75 (28.4)	54 (39.4)	66 (28.6)	35 (34.7)	126 (29.0)	127 (37.2)	267 (28.7)
6–10	22 (21.4)	59 (22.3)	28 (20.4)	53 (22.9)	28 (27.7)	114 (26.3)	78 (22.9)	226 (24.3)
11–15	19 (18.4)	47 (17.8)	14 (10.2)	36 (15.6)	13 (12.9)	70 (16.1)	46 (13.5)	153 (16.4)
16–20	9 (8.7)	21 (8.0)	16 (11.7)	25 (10.8)	5 (4.9)	48 (11.1)	30 (8.8)	94 (10.1)
21–25	3 (2.9)	21 (8.0)	8 (5.8)	19 (8.2)	7 (6.9)	25 (5.8)	18 (5.3)	65 (7.0)
26–30	1 (1.0)	11 (4.2)	2 (1.5)	7 (3.0)	3 (3.0)	18 (4.1)	6 (1.7)	36 (3.9)
> 30	3 (2.9)	8 (3.0)	2 (1.5)	8 (3.5)	2 (2.0)	10 (2.3)	7 (2.1)	26 (2.8)
Total	103 (100)	264 (100)	137 (100)	231 (100)	101 (100)	434 (100)	341 (100)	929 (100)

## Discussion

By analyzing medical records collected over 12 years, this pioneer study aims to analyze whether cancer histories of siblings should be an indicator for early opportunistic screening for early detection of cancer for such individuals. Overall, the most common cancer types and their proportions among male and female is consistent with the data of China from the 2018 Globocan [7].

More than half of Bro-pairs (55.3%) or Sis-pairs (51.1%) had cancer from same systemic origin, and more than a quarter of Bro-pairs (28.1%) and Sis-pairs (37.2%) developed the same type of cancer. Therefore, men whose brother is diagnosed with cancer should pay special attention to opportunistic screening for cancers from the same systemic origin, especially if the brother is diagnosed with lung cancer, liver cancer, stomach cancer, esophageal cancer or colorectal cancer. For women whose sister is diagnosed with cancer, likewise, she should consider participating in opportunistic screening for same-origin cancers, especially if the sister is diagnosed with breast cancer, lung cancer, cervical cancer, liver cancer, colorectal cancer or thyroid cancer. Although only 36.0% or 18.9% of siblings from Bro-Sis pairs developed same-origin cancers or same types of cancers, individual whose sibling is diagnosed with cancer should pay special attention to early screening of lung cancer or digestive system cancer if the sibling is diagnosed with these cancers.

Tobacco smoke (first- and second-hand) exposure, alcohol consumption and obesity are important risk factors for cancers worldwide [8]. Smoking is an especially important risk factor for lung cancer [9], while alcohol consumption is a vital risk factor for liver cancer [10]. In addition, chronic infection with hepatitis B virus or hepatitis C virus is the predominant cause of liver cancer [11], while infection with bacterium *helicobacter pylori* is the main risk factor for stomach cancer [12]. Dietary habit is also associated with several digestive system cancers. Low intake of fruits and vegetables increases risks of stomach cancer and esophageal cancer [13, 14], while high vegetables and fruits intake may protect individuals against esophagus cancer [15], colorectal cancer [16], and breast cancer [17]. Consumption of hot food and beverages is associated with an increased risk of esophageal cancer [18], while a high intake of dietary fiber, in particular cereal fiber and whole grains, reduces the risk of colorectal cancer [19, 20].

Special attention should be paid to thyroid cancer, as its incidence rate is increasing rapidly worldwide, especially in women, whose risk is 3 times higher than their male counterparts [7]. It is now the fourth most common cancer among Chinese women, which is similar to our study. Different than other cancers, Thyroid cancer is disproportionally diagnosed among younger population [21]. In this study, the median age of diagnosis of thyroid cancer is 48 years old, and 69.0% of incident cases occur in patients under 50 years. Established risk factors for thyroid cancer includes family history, obesity, alcohol and tobacco consumption, and ionizing radiation [21].

Therefore, changes in lifestyle and dietary habit play an important role in reducing the incidence of all cancers. As siblings share similar inherited genes, the changes in lifestyle are especially important for an individual if the sibling is diagnosed with cancer.

Hereditary tumor syndromes, which are caused by an inactivating mutation in a single crucial gene, increase the risk of cancers. Approximately 3–5% of patients with breast cancer and 8–17% of patients with ovarian cancer can attribute the cancer to germline pathogenic variants in the BRCA1 and BRCA2 genes [22–25], which is called the hereditary breast-ovarian cancer (HBOC) syndrome [24]. Individuals carrying mutations in BRCA1/BRCA are associated with a higher lifetime risk of up to 60–85% for breast cancer, and 17–39% for ovarian cancer by the age of 70 [26–28]. When an individual is found to have a germline BRCA1/BRCA2 mutation in HBOC or a DNA mis-match repair gene mutation in Lynch syndrome [29], the individual should inform their at-risk family members about the option of presymptomatic DNA testing. Due to the high cost of genetic testing and lack of informed consent from other family members, only a few participants underwent genetic testing, which reflects the low prevalence of genetic testing among Chinese population.

However, in this study, for siblings in sibling-pairs diagnosed with breast cancer, ovarian cancer or colorectal cancer, the majority of differences of ages-at-diagnosis was within 10 years. Among 77 Sis-pairs with breast cancer, the average age of diagnosis was 54.8. Among them, 54.5% of the ages-at-diagnosis differences between sisters were within 5 years, and 75.3% within 10 years. For 4 sis-pairs with ovarian cancer, the age differences were all within 10 years.

Across all sibling-pair groups, 28 sibling-pairs were diagnosed with colorectal cancer, and 32.1% of the pairs had age differences of less than 5 years, while 75.0% had age differences of less than 10 years.

Individuals with identified pathogenic variants in the BRCA1/BRCA2 gene can benefit from cancer risk-reducing strategies.

Considering that more than a quarter of Bro-pairs and Sis-pairs, and nearly 20% BroSis-pairs developed the same type of cancer, and that in these sibling-pairs with the same type of cancer, over 65% were diagnosed within the age difference of 10 years, genetic services are essential for individuals who has a sibling diagnosed with cancer.

Early diagnosis and treatment of cancer is very important to prolong the survival time of patients. In three sibling-pair groups, sibling-pairs with less than 5 years of age differences account for 35.9–40.8% of all pairs, while those with less than 10 years of age differences account for 61.1–62.8%. Usually, the development of cancer from precancerous lesions takes years, if not decades. Therefore, after a sibling has been diagnosed with cancer, the other sibling should be recommended to participate in opportunistic screening for early detection of cancer every year.

The most important strength of our study is its large sample size, which was collected during physical examination over 12 years. The study has several limitations. First, only individuals who underwent physical examination in one location were included in the study. Second, the ages of diagnosis were collected through surveys and face-to-face interviews, which may result in recall bias or information bias. However, the diagnosed age and cancer types of siblings were confirmed twice by first-degree relatives. In general, if the family member gets cancer, the first-degree relatives will take the information very seriously. Therefore, the recall bias is unlikely to have resulted in too much effect of the results. In addition, hospitals in China are organized according to a 3-tier system that recognizes a hospital’s ability to provide medical care, medical education, and conduct medical research. Based on this, hospitals are designated as Primary, Secondary or Tertiary institutions [30]. Tertiary hospitals round up the list as comprehensive or general hospitals at the city, provincial or national level. They are responsible for providing specialist health services, perform a bigger role with regard to medical education and scientific research and they serve as medical hubs providing care to multiple regions. In China, the patient tends to visited the tertiary hospital to confirm the disease, if he or she was diagnosed with the cancer in other level hospitals. Thus, cancer diagnosis information is relatively reliable. Third, no other risk factors or potential confounders were evaluated, which could cause confounding and selection bias. Finally, genetic testing for the siblings was not conducted. Further studies are needed to include multi-center samples, adjust for potential confounders and test for genomic DNA sequence.

In conclusion, by analyzing shared characteristics of sibling-pairs with cancers, this study concluded several recommendations for opportunistic screening for individuals whose siblings are diagnosed with cancers. When an individual’s sibling is diagnosed with cancer, the individual should consider participating in opportunistic screening each year, especially for lung cancer and digestive system cancers for both sexes. In addition, for female, breast cancer, cervical cancer and thyroid cancer should also be screened early. Furthermore, genetic services are essential for individuals who have siblings with cancer.

## Data Availability

The datasets used and analyzed during the current study are available from the corresponding author on reasonable request.

## Abbreviations

Bro: Brother
Sis: Sister
BroSis: Brother-and-sister
HBOC: Hereditary breast-ovarian cancer
